# Comparison of ventilation with second-generation supraglottic airway devices in a prospective randomized cadaver study

**DOI:** 10.1038/s41598-026-53005-5

**Published:** 2026-05-21

**Authors:** Frank Weilbacher, Nikolai Kaltschmidt, Marita Klein, Lisa Kaltschmidt, Harald Genzwürker, Erik Popp, Stephan Katzenschlager

**Affiliations:** 1 Medical Faculty Heidelberg, Department of Anaesthesiology, Heidelberg University Hospital, Im Neuenheimer Feld 420, 69120 Heidelberg, Germany; 2 Medical Faculty Heidelberg, Heidelberg University, Heidelberg, Germany

**Keywords:** Airway management, Supraglottic airway device, Emergency medicine, Laryngeal masks, Laryngeal tubes, Cadaver, Diseases, Health care, Medical research, Physiology

## Abstract

Second-generation supraglottic airway devices (SADs) are widely used alternatives to endotracheal intubation in both anesthesia and emergency airway management. The newly developed LT®evo represents a redesigned version of the established Laryngeal Tube Suction Disposable (LTS-D). This study compared the ventilatory performance of the LT®evo with endotracheal intubation and three other second-generation SADs— LTS-D, Ambu® AuraGain™ and i-gel® Plus—using a controlled human cadaver model. In this prospective, randomized experiment, six thawed adult cadavers were sequentially ventilated via an endotracheal tube (ET) and four SADs under standardized mechanical ventilation (tidal volume 7 mL/kg, PEEP 5 cmH₂O, respiratory rate 10/min). Airway pressure and flow were continuously recorded (sampling rate 200 Hz) to determine inspiratory tidal volume, pressure, flow, and calculated resistance. Endotracheal intubation using fiberoptics was attempted via LT®evo , AuraGain™ and i-gel® Plus. Data were analyzed using Kruskal–Wallis tests with Holm-corrected post-hoc pairwise comparisons. From six human cadavers (60–85kg estimated body weight), 30 complete ventilation datasets were obtained. Initial fiberoptic control of SAD placement showed optimal positioning in 13 of 24 insertions. During ventilation cycles, inspiratory tidal volumes of the LT®evo (0.49 [0.46–0.52]) were similar to those of ET, AuraGain™ and LTS-D and superior to those of the i-gel® Plus (0.31 L [0.27–0.33], p < 0.001). Peak inspiratory pressure and inspiratory resistance were higher in ET when compared to LT®evo and all other SADs. Endotracheal intubation using fiberoptics was successful in all attempts via LT®evo , AuraGain™ and i-gel® Plus. In this controlled cadaver study, the newly designed LT®evo demonstrated ventilatory performance comparable to that of the LTS-D and the Ambu® AuraGain^TM^ and was superior to the i-gel® Plus in delivering adequate tidal volumes. Additionally, LT®evo allowed for fiberoptic ET placement. Importantly, these results are specific to the cadaveric model and reflect technical variability in ventilation mechanics rather than clinical effectiveness.

Clinical trial number

German Clinical Trials Registry, DRKS00038309, on October 30^th^, 2025.

**Supplementary Information:**

The online version contains supplementary material available at 10.1038/s41598-026-53005-5.

## Introduction

Supraglottic airway devices (SAD) play a crucial role in airway management, providing an alternative to both bag-mask ventilation and endotracheal intubation in patients of all ages across various clinical scenarios, including emergencies. Various international and national Anaesthesia Guidelines for Airway Management recommend the use of SADs when mask ventilation and/or intubation are expected or found to be difficult or fail^1,2^. For cardiac arrest, the International Resuscitation Guidelines advise considering the use of supraglottic airway devices for advanced airway management^3^.

Following the introduction of the laryngeal mask airway in 1983 by Archie Brain^4^, a wide range of supraglottic airway devices has been developed. So-called ‚second generation‘ SADs with improved seal and an additional gastric channel are currently recommended, especially for emergency settings in unfasted patients^1,2^. Some devices can also be used as a conduit for fiberoptic intubation once adequate ventilation is ensured.

Whenever new devices are introduced, early evaluation and comparison with currently available products is desirable. The Laryngeal Tube, a reusable silicone device, was first introduced in 2000^5,6^. A first version with a gastric drain channel, the Laryngeal Tube Suction, became available in 2002^7^. The development of paediatric sizes necessitated a redesign of the device, and the trend toward single-use devices led to the LTS-D that has been available since 2005. The new Laryngeal Tube LT®evo (VBM Medizintechnik, Germany) is a completely redesigned version of the LTS-D. The following major design changes have been made: An oval cross-section of the tube and modified shapes of the proximal and distal cuffs are intended to improve positioning. Epiglottis deflectors are designed to reduce the risk of the airway being obstructed by the epiglottis. The ventilation channel has been enlarged and the ventilation opening has been adapted to allow endotracheal intubation via the LT evo using a flexible fiberoptic, which is not possible with the LTS-D. Additionally, softer, more flexible materials have been incorporated.

This study aimed to compare ventilation with the newly designed LT®evo to the following well established airway devices: (1) LTS-D as the predecessor of LT®evo and as a laryngeal tube type SAD. (2) Ambu® AuraGain^TM^ (Ambu A/S, Denmark) as a laryngeal mask type SAD with inflatable cuff available since 2014^8^. (3) i-gel^®^ Plus (Intersurgical, United Kingdom), as a laryngeal mask type SAD with a non-inflatable seal composed of a thermoplastic elastomer, first introduced in 2007^9^, with the redesigned version, i-gel^®^ Plus, being introduced in 2021^10^. (4) Endotracheal tube (ET) as the current gold standard of invasive airway management.

The feasibility of second-generation SADs for ventilation is of paramount importance for routine clinical use and emergency care. However, there is no data on how LT®evo as a complete redesign compares to currently used SADs and ET with regards to ventilation performance. The present study was designed to fill this knowledge gap by comparing LT®evo against three widely used second-generation SADs and ET in adult cadavers. At the initiation of this study, the LT®evo lacked CE certification; therefore, a cadaveric model was chosen.

## Methods

This was a prospective, randomized, experimental study conducted using a human cadaver ventilation model. Six thawed, non-embalmed human cadavers were used to evaluate ventilatory performance with different airway devices. Each cadaver was thawed at room temperature for two days before the study.

All procedures were conducted in accordance with institutional guidelines and German legislation governing the use of post-mortem human tissue in research. Written consent for scientific use was obtained during the donors’ lifetime by the Department of Anatomy at Heidelberg University. This study was approved by the Heidelberg Ethics Committee (S186-2025) on May 8^th^, 2025, and registered in the German Clinical Trials Registry (DRSK ID: DRKS00038309) on October 30^th^, 2025. After the study, the cadavers were used in a training course for invasive emergency techniques^11^. The reporting of this study follows the *Reporting ChAracteristics of cadaver training and sUrgical studies: The CACTUS guidelines*^12^.

### Airway device selection, insertion and ventilation protocol

Each cadaver was initially intubated by a board-certified anesthesiologist using direct laryngoscopy and a standard endotracheal tube (RÜSCH® Super SafetyClear™ with cuff, Teleflex, USA) with 7.5mm inner diameter (ID) for female and 8.0mm ID for male cadavers. During this step, the Cormack–Lehane grade of glottic visualization was assessed and documented for each specimen. This was done to establish a baseline reference condition for airway patency and access.

Subsequently, the cadaver was ventilated with the ET and then underwent sequential insertion of the remaining four airway devices, following a randomized design to minimize order effects.

Randomization was performed a priori by two members of the study group (SK and NK). Using a random number sequence generated with Microsoft Excel, one supraglottic airway device was matched with one cadaver. The remaining sequence was fixed and defined before the study started.

The supraglottic airway devices used in this study were:

Laryngeal Tube LT®evo (VBM Medizintechnik, Germany).

Laryngeal Tube Suction Disposable (LTS-D, VBM Medizintechnik, Germany).

Ambu® AuraGain^TM^ (Ambu A/S, Denmark).

The i-gel^®^ Plus (Intersurgical, United Kingdom)^13^.

The laryngeal mask types are routinely used in our department. All four products are single-use, second-generation SADs (supplement Figures 1-4). No prewarming of the airway devices was performed.

The size of each supraglottic airway device was chosen according to the respective manufacturer’s recommendations. After inserting the SGA according to the respective instructions for use, the position was confirmed using a fiberoptic scope (Ambu® aScope^TM^, Ambu A/S, Denmark). Repositioning was performed under fiberoptic guidance if the SGA was not ideally placed. Two anesthesia specialists performed the repositioning according to their clinical expertise; no standardized protocol was used.

I-gel^®^ Plus does not use an inflatable cuff, so no cuff pressure management was provided. LTS-D and LT®evo were inserted, and the cuffs were inflated using the inflation volumes marked on the dedicated syringes supplied with the devices. Ambu Aura Gain is delivered with a partially inflated cuff and was inserted without manipulation of cuff pressure and volume. Following this insertion procedure, initial cuff pressure (CP 1) was recorded for every device using a commercial manometer (Universal Cuff Manometer, VBM Medizintechnik, Germany). This device was chosen because it is used at the study’s author’s institution. Next, cuff pressure in all SADs with a cuff was set to 60cmH2O, the highest value within the manufacturer’s specifications for the respective devices (CP 2).

For ET, cuff pressure was set to 25 cmH2O after insertion, according to clinical standards, and no further measurements or adjustments were performed.

Before ventilation cycles, fiberoptic intubation was attempted through the LT®evo, i-gel® Plus, and Ambu® Aura Gain^TM^, and success rates were recorded. The LTS-D does not allow tracheal tube insertion through the ventilation channel.

Subsequently, a volume-controlled ventilation mode was used (tidal volume: 7 mL/kg estimated body weight, PEEP: 5 cmH₂O, respiratory rate: 10/min, I:E ratio: 1:2, Pmax: 50 mbar) to simulate controlled mechanical ventilation. With each device, each cadaver was ventilated for two minutes. In cases where ideal fiberoptic placement was achieved but sufficient ventilation could not be achieved, no further manipulations were undertaken.

After 2 minutes of ventilation, cuff pressure was measured again to document possible passive changes in cuff pressure during ventilation (CP 3).

Afterwards, the next SAD device was used (Supplement Figure 5). Due to the devices’ single-use nature, new, original packaged products were used for each round.

### Endpoints

The primary endpoint was defined as successful ventilation, as determined by the criteria listed below. This was determined over 2 minutes, during which volume-controlled ventilation was performed.

Each endpoint was analyzed individually.

- inspiratory tidal volume
- inspiratory airway pressure
- inspiratory flow
- calculated inspiratory resistance

Inspiratory tidal volume was selected as a primary endpoint because it represents the volume delivered by the ventilator and transmitted through the airway device, independent of expiratory leakage or circuit-related losses. This approach has been used in prior cadaveric and mechanical ventilation studies to compare supraglottic airway devices under controlled ventilation conditions^14^. The threshold for adequate inspiratory tidal volume was defined as 6 mL/kg of body weight according to current recommendations^15^.

Secondary endpoints are defined as (a) first attempt success, and (b) initial cuff pressure.

First-attempt success is defined as placement of the SAD with fiberoptic confirmation.

No formal analysis of Cormack–Lehane grade was undertaken, as grading was performed at the cadaver level and showed insufficient variability for a meaningful comparison with repeated device-level first-attempt success outcomes.

### Data acquisition and processing

Airway pressure and flow signals were recorded continuously using a calibrated differential pressure and flow sensor system (SFM 3304-D, Sensirion AG, Switzerland), which was connected in line between the ventilator and the airway device. This sensor is already calibrated and does not need further recalibration. After each device, the single-use sensor was replaced. Data were exported as CSV files at a sampling rate of 200 Hz. For each ventilation cycle, key respiratory parameters were extracted, including inspiratory tidal volume (Vt), inspiratory flow, inspiratory airway pressure, and calculated inspiratory resistance (defined as peak inspiratory pressure divided by peak flow during inspiration).

The raw flow data were integrated over time to calculate tidal volume per respiratory cycle. Inspiratory pressure was defined as the peak airway pressure during the inspiratory phase, and inspiratory flow as the peak flow above baseline. The inspiratory resistance was calculated as the quotient of peak inspiratory pressure (converted from Pascal to mmHg) divided by peak inspiratory flow (in slm). Each parameter was extracted for at least five stable consecutive breaths per device and averaged per subject. Although airway resistance is commonly expressed in cmH₂O/L/s, values were reported in mmHg/slm to reflect the native pressure and flow units used for signal acquisition and analysis; conversion to cmH₂O/L/s can be performed using this conversion: 1 mmHg/slm ≈ 0.816 cmH₂O/L/s.

### Statistical analysis

Data are presented as median and interquartile range (IQR) for each device. Baseline characteristics or prior diseases and cause of death for the human cadavers were not available. Bodyweight was estimated by five members of the study team in consensus. Comparisons between devices were performed using the Kruskal–Wallis test, followed by pairwise Mann–Whitney U tests for post hoc analysis, corrected for multiple testing using the Holm method. Estimated median differences are presented, along with corresponding 95% confidence intervals (CIs). Statistical significance was defined as p < 0.05. Because of the exploratory nature of the study, no prior power analysis was performed.

All analyses were performed using Python (v3.10) and the libraries NumPy, SciPy, Pandas, and Matplotlib.

## Results

### Descriptive ventilation characteristics

The human cadavers consisted of six adults (four male, two female), with an estimated body weight between 60 and 85 kg, resulting in a mean calculated target tidal volume of 0.502 L. Calculated mean lower threshold for adequate ventilation was 0.43 L. The Cormack-Lehane grade was 1 in four cadavers and 2 in the remaining two. Initial fiberoptic control of glottic view after SAD placement showed optimal view in 13 of 24 insertions. In the remaining attempts, the SAD’s position had to be manually corrected using fiberoptic guidance (Table 1).

**Table 1:** Human cadavers and supraglottic airway devices baseline characteristics.

Cadaver #	Sex	Estimated BW [kg]	C/L	Device	FAS	Cuff pressure initially (CP 1) [cmH_2_O]	Cuff pressure after ventilation cycle (CP 3) [cmH_2_O]
1	Male	60	1	LT®evo	No	20	40
				Aura Gain^TM^	No	40	40
				LTS-D	No	70	50
				i-gel® Plus	Yes	–	–
2	Male	85	1	i-gel® Plus	No	–	–
				LT®evo	Yes	>120	60
				Aura Gain^TM^	Yes	112	65
				LTS-D	No	75	55
3	Male	70	1	LTS-D	No	75	50
				i-gel® Plus	Yes	–	–
				LT®evo	No	40	34
				Aura Gain^TM^	No	80	55
4	Male	65	1	Aura Gain^TM^	Yes	75	50
				LTS-D	Yes	88	60
				i-gel® Plus	Yes	–	–
				LT®evo	No	30	40
5	Female	75	2	LT®evo	Yes	60	40
				Aura Gain^TM^	Yes	80	60
				LTS-D	No	120	60
				i-gel® Plus	No	–	–
6	Female	75	2	i-gel® Plus	Yes	–	–
				LT®evo	Yes	75	40
				Aura Gain^TM^	Yes	85	50
				LTS-D	Yes	120	60

The i-gel® Plus lacks cuff pressure due to its non-inflatable thermoplastic elastomer design. BW = Body weight; FAS = First attempt success; C/L = Cormack Lehane grade. A total of 30 complete ventilation datasets were obtained from six human cadaveric models, each of which was ventilated sequentially using five different airway devices (ET and four SADs).

### Ventilation parameters

Table 2 provides an overview of median values and interquartile ranges (IQR) for each airway device. LT®evo, as well as LTS-D, Aura Gain^TM^ and ET, produced adequate mean tidal volumes above 6 ml/kg of body weight. In contrast, mean inspiratory tidal volumes achieved by the i-gel® Plus (0.31 L [0.27–0.33]) did not reach the defined threshold for adequacy of 0.43 L.

**Table 2:** Median [Q1–Q3] values of ventilation parameters per airway device.

Device	Tidal Volume [L]	Flow [slm]	Pressure [mmHg]	Resistance [mmHg/slm]
LT^®^evo	0.49 [0.46–0.52]	11.8 [10.3–12.8]	9.7 [8.8–10.7]	0.82 [0.75–0.90]
LTS-D	0.51 [0.50–0.54]	11.7 [11.0–12.3]	9.6 [8.9–10.3]	0.82 [0.78–0.87]
Aura GainTM	0.51 [0.49–0.53]	12.0 [11.2–13.1]	10.5 [9.7–11.2]	0.89 [0.84–0.93]
i-gel^®^ Plus	0.31 [0.27–0.33]	8.8 [8.3–9.3]	9.5 [8.9–10.1]	1.09 [1.03–1.15]
ET	0.51 [0.47–0.57]	12.0 [10.9–13.2]	16.8 [15.4–18.1]	1.41 [1.33–1.51]

ET = endotracheal tube, slm = standard liters per minute.

### Airway device comparisons

Table 3 shows the mean differences of ventilation parameters when comparing LT®evo to the other airway devices. There were no differences in inspiratory tidal volumes compared to LTS-D, Aura Gain^TM^, and ET. Compared to the i-gel® Plus, the LT®evo delivered higher tidal volumes. Inspiratory pressures with the LT®evo were higher than with the LTS-D, Aura GainTM, and ET. Inspiratory resistance was higher in ET and marginally lower in LTS-D. Although the differences to LTS-D are statistically significant, clinical significance remains questionable. The complete comparisons between the devices are presented in Supplement Table 1.

**Table 3:** Median differences (95% CI) values of ventilation parameters.

Parameter	Device A	Device B	Median Difference (95% CI)	p-value
Tidal Volume (L)	LT^®^evo	LTS-D	−0.008 (−0.111; 0.099)	1.0
Tidal Volume (L)	LT^®^evo	Aura Gain^TM^	−0.005 (−0.088 to 0.098)	1.0
Tidal Volume (L)	LT^®^evo	i-gel® Plus	0.191 (0.106 to 0.301)	<0.001
Tidal Volume (L)	LT^®^evo	ET	−0.015 (−0.066 to 0.035)	0.742
Inspiratory Pressure (mmHg)	LT^®^evo	LTS-D	0.96 (0.61 to 1.34)	<0.001
Inspiratory Pressure (mmHg)	LT^®^evo	Aura Gain^TM^	0.38 (0.04 to 0.73)	0.028
Inspiratory Pressure (mmHg)	LT^®^evo	i-gel® Plus	0.34 (−0.02 to 0.71)	0.071
Inspiratory Pressure (mmHg)	LT^®^evo	ET	−6.31 (−9.41 to −3.87)	<0.001
Inspiratory Resistance (mmHg/slm)	LT^®^evo	LTS-D	0.08 (0.06 to 0.11)	<0.001
Inspiratory Resistance (mmHg/slm)	LT^®^evo	Aura Gain^TM^	0.03 (−0.45 to 0.34)	1.0
Inspiratory Resistance (mmHg/slm)	LT^®^evo	i-gel® plus	−0.02 (−0.06 to 0.01)	0.19
Inspiratory Resistance (mmHg/slm)	LT^®^evo	ET	−0.59 (−0.68 to -0.50)	<0.001

### Pressure–flow loop analysis

**Fig. 1:**
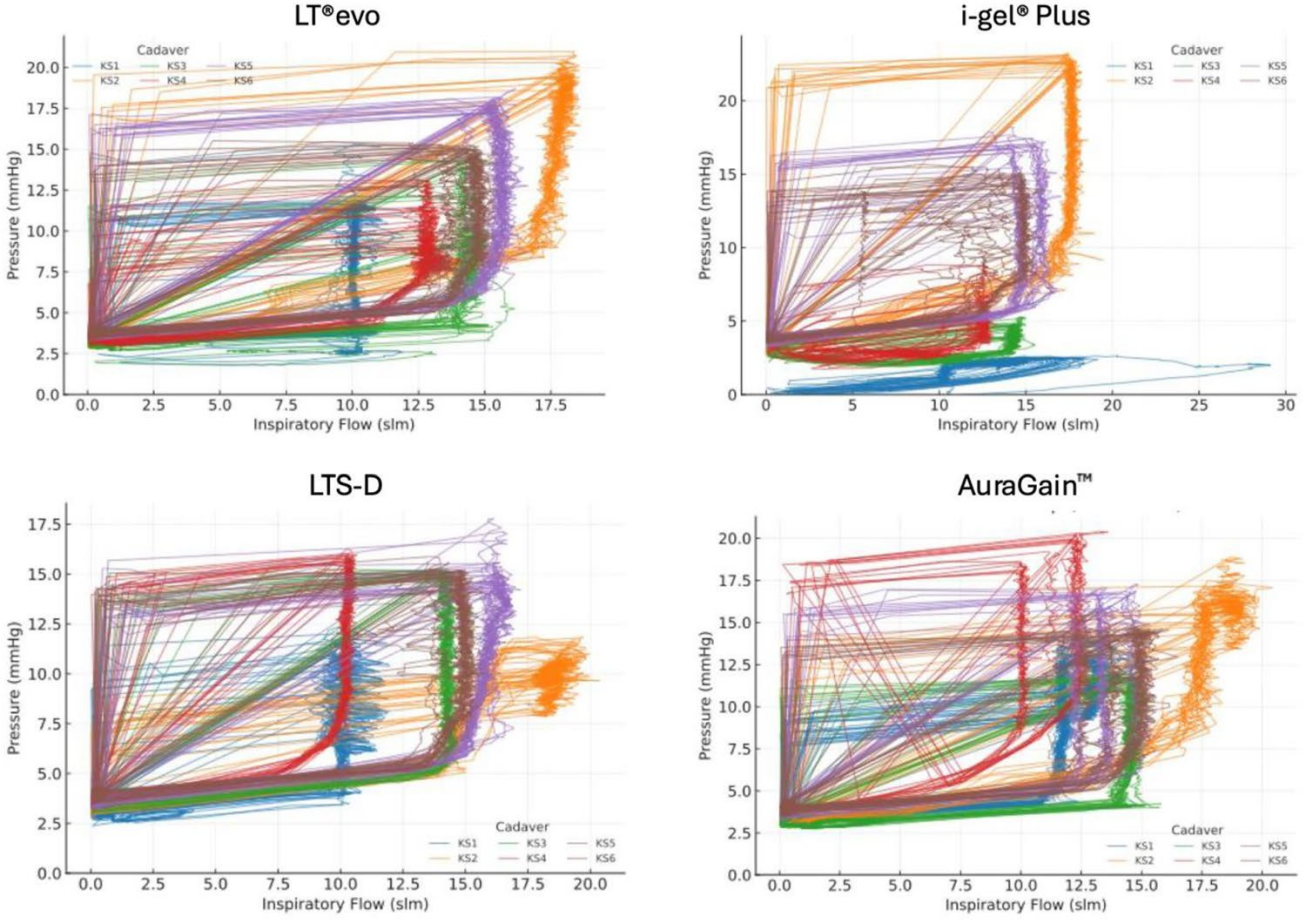
Representative pressure–flow loops for each airway device. A higher loop slope indicates increased resistance. The i-gel® Plus showed the flattest profile, corresponding to lower tidal volumes and inspiratory flow. Each color corresponds to one cadaver. KS 1–6 indicates the individual human cadaver.

When compared to the LT evo and to the other airway devices, the i-gel® Plus exhibits lower inspiratory flow and a flattened pressure rise, which supports the quantitative findings (Fig. 1).

### Sensitivity analysis

To assess the robustness of the findings, a sensitivity analysis was conducted restricted to the three cadavers in which the i-gel® produced technically comparable pressure–flow recordings, although delivered tidal volumes remained below the ventilator-set target in two cadavers across all devices (Supplement Tables 2 and 3).

### Secondary endpoints

After correct placement and ventilation, fiberoptic endotracheal intubation through the respective SAD was possible via LT®evo, AuraGain™ and i-gel® Plus in all attempts.

Pairwise comparisons of first-attempt success between supraglottic airway devices were performed using two-sided Fisher’s exact tests. No statistically significant differences were observed between any device pairs (all p ≥ 0.56), although numerically higher success rates were noted for Aura Gain™ and i-gel® Plus compared with LTS-D.

After exclusion of i-gel® Plus and recoding values >120 mmHg as 120 mmHg, initial cuff pressures were lowest for LT®evo (median 50.0 [IQR 35.0–67.5] mmHg), intermediate for AuraGain™ (80.0 [77.5–82.5] mmHg), and highest for LTS-D (81.5 [75.0–104.0] mmHg). In exploratory paired analyses, there were no statistically significant differences between the three devices (LT®evo vs AuraGain™, p=0.063; LT®evo vs LTS-D, p=0.125; or AuraGain™ vs LTS-D, p=0.438).

## Discussion

This study compared the newly designed LT®evo to four other airway devices—LTS-D, Ambu® AuraGain^TM^, i-gel® Plus and endotracheal tube— regarding their ventilatory performance in a controlled cadaver model using high-resolution pressure and flow measurements.

According to our results, LT®evo can deliver adequate tidal volumes with modest inspiratory pressures. Inspiratory tidal volumes delivered via LT®evo were comparable to those of ET and the other SGAs, with the exception of the i-gel® Plus.

In pairwise comparison, LT®evo and LTS-D demonstrated comparable ventilation performance. Inspiratory pressure was marginally lower in the LTS-D, possibly due to design changes. When evaluated in paediatric patients by Katzenschlager et al., different success rates in the first insertion attempt were observed between the LTS-D and Ambu® AuraGain^TM^^16^. Further studies will be necessary to evaluate whether similar effects are present for the LT®evo.

Compared to the Ambu® AuraGain^TM^, LT®evo’s performance was also broadly similar. While providing comparable tidal volumes, AuraGain^TM^ exhibited slightly higher inspiratory pressures, possibly due to its bulkier design or differences in cuff compliance^17^.

With regards to the i-gel® Plus , the observed approximately 200ml difference in tidal volume compared to the LT®evo and the other airway devices represents a significant difference in ventilatory performance under standardized experimental conditions, reflecting a relevant mechanical and technical variance rather than a direct clinical effect. These results differ from a recent network meta-analysis, which found that the i-gel® performed significantly better in human trials^18^. The observation of lower inspiratory tidal volumes with the i-gel® Plus in the primary analysis warrants careful interpretation. Under volume-controlled ventilation, the inspiratory tidal volume is expected to be similar across devices if ventilation is fully effective. However, in this cadaver model, delivered inspiratory volume reflects not only ventilator settings but also device-specific factors such as seal stability, anatomical conformity, and dynamic leakage during inspiration. Notably, inspiratory airway pressures with the i-gel® Plus did not consistently reach pressure limits, suggesting that reduced inspiratory tidal volume was unlikely to be driven by pressure restriction alone. Instead, these findings likely reflect incomplete transmission of the set tidal volume due to subtle inspiratory leakage or transient loss of seal integrity, phenomena that may not be fully captured by peak pressure measurements alone.

Our results generally align with current evidence regarding second-generation SADs, though performance of different SADs varied across previous studies in different settings. In elective laparoscopic surgery, two studies have demonstrated that SADs maintain tidal volume and normocapnia comparable to those of endotracheal tubes, with the added benefit of lower peak inspiratory pressures and improved dynamic compliance^19,20^. Similarly, the i-gel required lower inspiratory pressures in elective daycare surgery patients^21^. In emergency situations, SADs delivered lower tidal volumes and lower inspiratory pressures than endotracheal tubes during cardiopulmonary resuscitation, with greater leak and inter-device variability^14,22^. In contrast to our findings, the i-gel outperformed the AuraGain™ in sealing and pressure maintenance in another cadaver study^22^. In a study by Russo et al., the fiberoptically assessed position was more frequently suboptimal with the LTS-D^23^, as observed in our study.

ET, as the clinical gold standard for invasive airway management, delivered similar tidal volumes to LT®evo but required higher inspiratory pressures than all SGAs, likely due to increased resistance. This effect is well-established across a variety of ventilation settings^24^.

In a sensitivity analysis restricted to cadavers with technically interpretable pressure–flow recordings, no significant differences in inspiratory tidal volume were observed between supraglottic airway devices. Notably, although pressure–flow loops indicated effective ventilation, delivered tidal volumes remained below the ventilator-set target in two cadavers across all devices, including the endotracheal tube. This finding suggests that the reduced absolute tidal volumes in this subset were driven by cadaver-specific factors rather than device-related differences. Consequently, when an effective seal is achieved, the i-gel® Plus appears capable of delivering tidal volumes comparable to those of other second-generation supraglottic airway devices, while differences observed across the overall cohort are likely attributable to variability in seal performance and ventilation conditions.

From a clinical perspective, our findings are directly relevant for airway management decisions in anesthesia and prehospital care. Although ET intubation remains the gold standard, it requires greater expertise and time to secure the airway, potentially delaying ventilation in urgent situations^2^. SADs provide quick insertion and adequate ventilation, making them vital tools in rescue protocols and the management of difficult airways^25,26^. In contrast to its predecessor LTS-D, the newly developed LT®evo offers the option of fiberoptic intubation, adding a technical advancement that could improve airway management in general anesthesia, pre-hospital emergency medicine, or other emergency situations. When compared to its predecessor in our cadaver model, the LT®evo demonstrated similar findings across all ventilation parameters. These results need to be corroborated in clinical trials, as human cadaver trials have certain limitations. Importantly, as with AuraGain™ and i-gel® Plus, placing an endotracheal tube using fiberoptic guidance was possible in all attempts via LT®evo. Given that ventilation performance was comparable to that of LTS-D, the clinically most important advancement of the LT®evo may lie in its ability to serve as a reliable bridge to definitive airway management, which also needs to be confirmed in clinical trials.

It is essential to acknowledge the limitations of this study. (A) Cadaveric specimens lack muscle tone, spontaneous breathing efforts, secretions, and blood flow, all of which affect SAD performance in living subjects. This also restricts the ability to assess PaO2, PaCO2, and EtCO2. SAD placement required fiberoptic correction in approximately 50% of cases. As fiberoptic correction is typically not used in clinical or emergency settings, this study examined the devices’ performance under optimal conditions; performance after blind insertion without correction may vary. (B) Airway tissues may exhibit altered compliance due to post-mortem changes, preservation methods and temperature, which can affect device fit and seal. This may be a significant concern regarding the ventilation performance of the i-gel® Plus. Benchtop studies have shown only minor variation of hardness and resilience values of the thermoplastic elastomer between room temperature and body temperature, and the device is also constructed to work in hypothermic patients^27^. Nevertheless, when compared to living patients, thermal conditions in this cadaver model may have been suboptimal for the use of the i-gel® Plus. This thermal bias may have caused reduced seal and could explain the lower tidal volumes observed in our study. Our results, especially with regards to the i-gel® Plus, may therefore not be readily applicable to living patients. Despite these limitations, cadaver models provide a consistent environment for measuring mechanical ventilation parameters, free from factors like patient movement and anesthesia-related variability. (C) Additionally, while our pressure-flow loops and calculated resistances offer detailed insights into mechanical ventilation, they do not account for issues such as aspiration risk, device malposition, or ease of use—elements that are important when evaluating an airway device comprehensively. (D) From a study design perspective, the protocol, which involved a randomized device order and standardized ventilation settings, enabled strong within-subject comparisons. (E) The consensus-based weight estimation by five team members may have affected the calculation of the target tidal volume, and thus the interpretation of the absolute tidal volume values. (F) The Ambu® AuraGain^TM^ was chosen as one representative of a wide variety of laryngeal mask airway devices available on the market. Results may not be applicable to other commonly used laryngeal masks, such as the LMA® Supreme™.

Nevertheless, the sample size was limited by the number of available cadaver donations. Although sufficient for exploratory and comparative analyses, larger in vivo studies are necessary to determine the clinical relevance of the observed differences.

## Conclusion

In this controlled cadaver study, the newly designed LT®evo demonstrated ventilatory performance comparable to that of the LTS-D and the Ambu® AuraGain^TM^ while performing superior to the i-gel® Plus in delivering adequate tidal volumes. Additionally, LT®evo allowed for fiberoptic ET placement in all attempts. Importantly, these results are specific to the cadaveric model and reflect technical variability in ventilation mechanics rather than clinical effectiveness. Consequently, prospective clinical trials remain essential to evaluate in vivo performance, particularly regarding sealing behavior at physiological temperatures, interaction with airway secretions, and potential mucosal effects.

## Supplementary Information

Supplementary Information.

## Data Availability

The datasets used and/or analyzed during the current study are available from the corresponding author on reasonable request.
