# Supplementary material for: Comparison of ventilation with second-generation supraglottic airway devices in a prospective randomized cadaver study
Source: Sci Rep. 2026 May 21;16:15873. doi: 10.1038/s41598-026-53005-5 (PMC13195101; doi:10.1038/s41598-026-53005-5)
Supplement: Supplementary file 1 — Supplementary Information. [file 41598_2026_53005_MOESM1_ESM.docx]

Supplementary Material to

Comparison of ventilation with second-generation supraglottic airway devices in a prospective randomized cadaver study

Frank Weilbacher^1^, Nikolai Kaltschmidt^1^, Marita Klein^1^, Lisa Kaltschmidt^1^, Harald Genzwürker^2^, Erik Popp^1^, Stephan Katzenschlager^1,#^

1 Department of Anaesthesiology, Heidelberg University, Medical Faculty Heidelberg, Heidelberg, Germany

2 Heidelberg University, Medical Faculty Heidelberg, Heidelberg, Germany

# correspondence to

Dr. med. Stephan Katzenschlager

Department of Anaesthesiology, Heidelberg University Hospital, Germany

Im Neuenheimer Feld 420, 69120 Heidelberg, Germany

E-mail: [stephan.katzenschlager@med.uni-heidelberg.de](mailto:maximilian.dietrich@med.uni-heidelberg.de)

Phone: +49 6221 56 39683

Table of Contents

[Supplemental Figure 1 - Laryngeal Tube LT®evo (VBM Medizintechnik, Germany) 3](#_Toc228973801)

[Supplemental Figure 2 - Ambu® AuraGain^TM^ (Ambu A/S, Denmark) 4](#_Toc228973802)

[Supplemental Figure 3 - Laryngeal Tube Suction Disposable (LTS-D, VBM Medizintechnik, Germany) 5](#_Toc228973803)

[Supplemental Figure 4 - i-gel^®^ Plus (Intersurgical, United Kingdom) 6](#_Toc228973804)

[Supplemental Figure 5 – Randomization order 7](#_Toc228973805)

[Supplement Table 1 – Comparison of inspiratory pressure, tidal volume, and inspiratory resistance 8](#_Toc228973806)

[Supplement Table 2 – Sensitivity analysis for KS 2, 5, and 6 for inspiratory pressure, tidal volume, flow, and inspiratory resistance 10](#_Toc228973807)

[Supplement Table 3 – Sensitivity analysis for KS 2,5, and 6 Comparison of inspiratory pressure, tidal volume, and inspiratory resistance 11](#_Toc228973808)

# Supplemental Figure 1 - Laryngeal Tube LT®evo (VBM Medizintechnik, Germany)


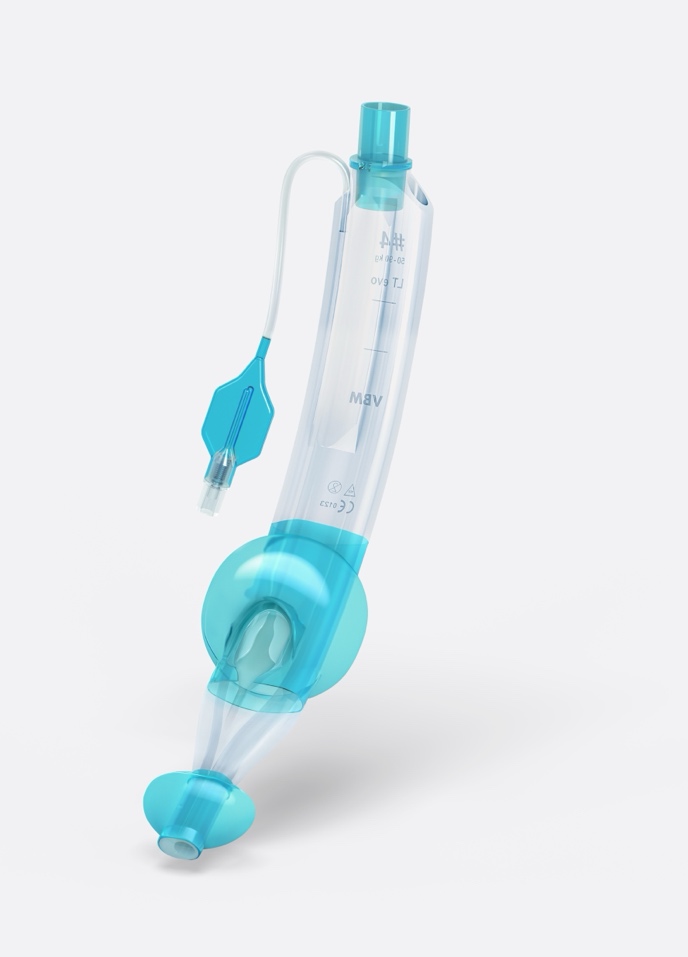


Used with permission from VBM Medizintechnik, Germany

# Supplemental Figure 2 - Ambu® AuraGain^TM^ (Ambu A/S, Denmark)


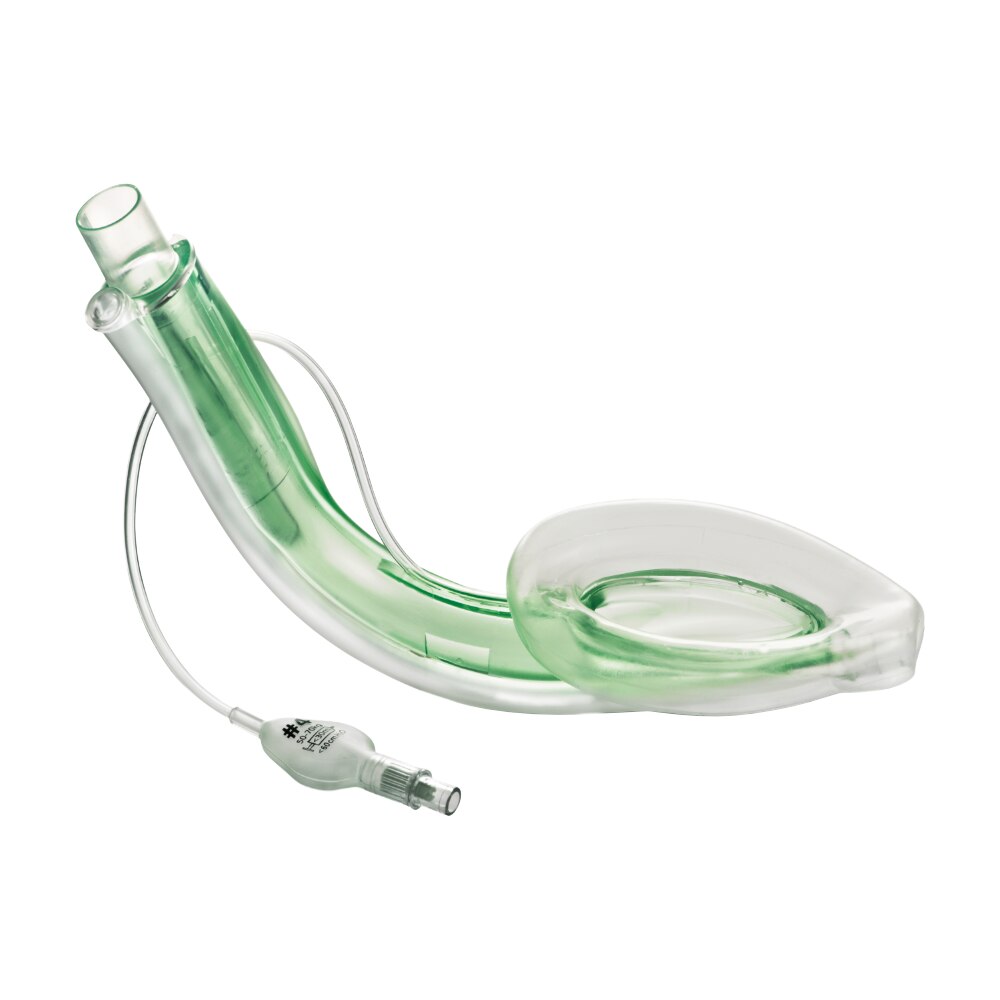


Open access from: https://www.ambu.de/atemwegsmanagement-anasthesie/larynxmasken/produkt/ambu-auragain-disposable-laryngeal-mask

# Supplemental Figure 3 - Laryngeal Tube Suction Disposable (LTS-D, VBM Medizintechnik, Germany)


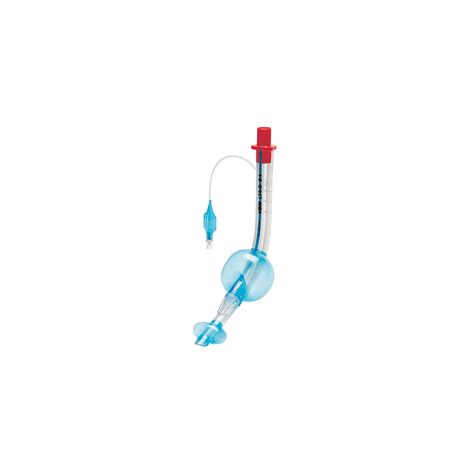


Used with permission from VBM Medizintechnik, Germany

# Supplemental Figure 4 - i-gel^®^ Plus (Intersurgical, United Kingdom)


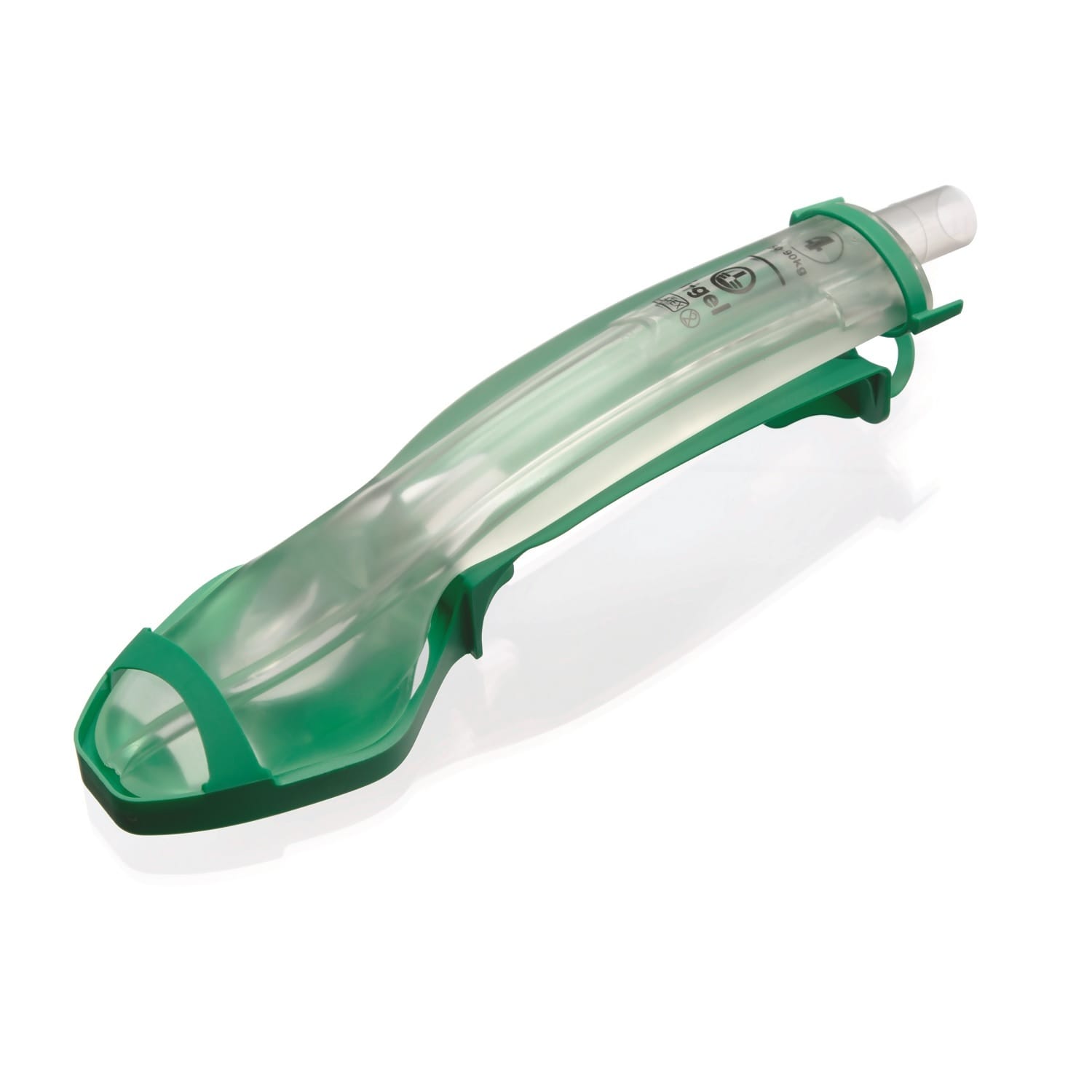


Open access from: https://www.intersurgical.de/info/igel-plus

# Supplemental Figure 5 – Randomization order


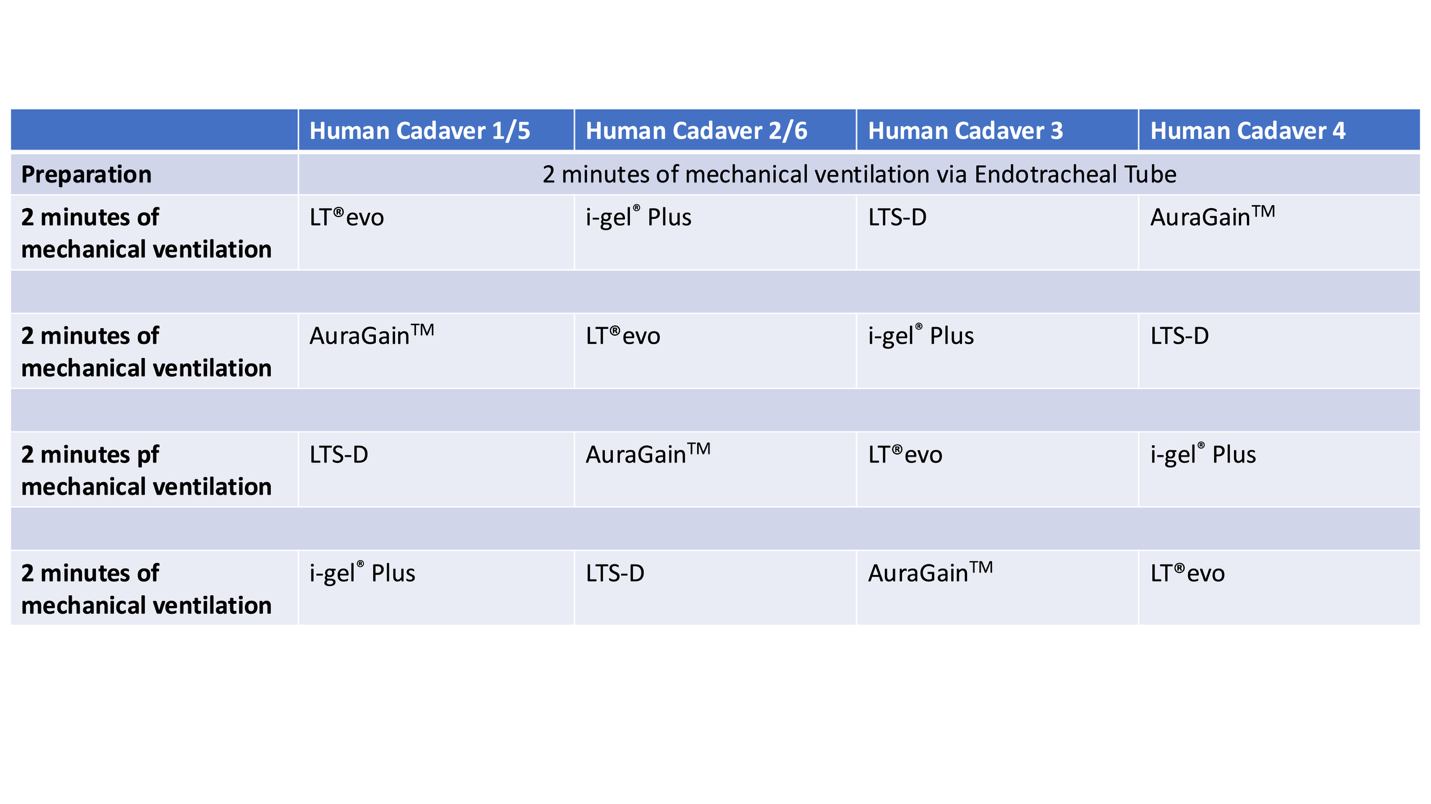


Caption: This figure demonstrates the randomization order for the study including predefined cross-over sequences.

# Supplement Table 1 – Comparison of inspiratory pressure, tidal volume, and inspiratory resistance

| **Parameter** | **Device A** | **Device B** | **Median Difference (95% CI)** | **p-value** |
| --- | --- | --- | --- | --- |
| Inspiratory Pressure (mmHg) | Aura Gain^TM^ | LTS-D | 0.58 (0.22 to 0.97) | 0.002 |
| Inspiratory Pressure (mmHg) | Aura Gain^TM^ | i-gel® Plus | -0.04 (-0.31 to 0.41) | 0.84 |
| Inspiratory Pressure (mmHg) | ET | Aura Gain^TM^ | 6.28 (5.66 to 6.90) | <0.001 |
| Inspiratory Pressure (mmHg) | ET | LTS-D | 7.10 (6.55 to 7.66) | <0.001 |
| Inspiratory Pressure (mmHg) | ET | i-gel® Plus | 4.56 (3.79 to 5.35) | <0.001 |
| Inspiratory Pressure (mmHg) | ET | LT^®^evo | 6.31 (3.87 to 9.41) | <0.001 |
| Inspiratory Pressure (mmHg) | LT^®^evo | LTS-D | 0.96 (0.61 to 1.34) | <0.001 |
| Inspiratory Pressure (mmHg) | LT^®^evo | i-gel® Plus | 0.34 (-0.02 to 0.71) | 0.071 |
| Inspiratory Pressure (mmHg) | Aura Gain^TM^ | LT^®^evo | -0.38 (-0.73 to -0.04) | 0.028 |
| Inspiratory Pressure (mmHg) | LTS-D | i-gel® Plus | -0.63 (-1.07 to -0.19) | 0.004 |
| Tidal Volume (L) | Aura Gain | i-gel® Plus | 0.197 (0.104 to 0.309) | <0.001 |
| Tidal Volume (L) | ET | Aura Gain^TM^ | 0.002 (-0.042 to 0.047) | 0.932 |
| Tidal Volume (L) | ET | LT^®^evo | 0.015 (-0.035 to 0.066) | 0.742 |
| Tidal Volume (L) | ET | LTS-D | 0.012 (-0.038 to 0.064) | 0.785 |
| Tidal Volume (L) | ET | i-gel® Plus | 0.206 (0.114 to 0.319) | <0.001 |
| Tidal Volume (L) | LT^®^evo | i-gel® Plus | 0.191 (0.106 to 0.301) | <0.001 |
| Tidal Volume (L) | LTS-D | i-gel® Plus | 0.194 (0.109 to 0.306) | <0.001 |
| Tidal Volume (L) | LT^®^evo | LTS-D | -0.008 (-0.111; 0.099) | 1.0 |
| Tidal Volume (L) | Aura Gain^TM^ | LT^®^evo | 0.005 (-0.098 to 0.088) | 1.0 |
| Tidal Volume (L) | Aura Gain^TM^ | LTS-D | -0.002 (-0.119 to 0.074) | 1.0 |
| Inspiratory Resistance (mmHg/slm) | ET | i-gel® Plus | 0.34 (0.17 to 0.37) | <0.001 |
| Inspiratory Resistance (mmHg/slm) | ET | Aura Gain^TM^ | +0.58 (0.31 to 1.01) | <0.001 |
| Inspiratory Resistance (mmHg/slm) | ET | LT^®^evo | 0.59 (0.50 to 0.68) | <0.001 |
| Inspiratory Resistance (mmHg/slm) | ET | LTS-D | 0.59 (0.47 to 0.70) | <0.001 |
| Inspiratory Resistance (mmHg/slm) | LT^®^evo | LTS-D | 0.08 (0.06 to 0.11) | <0.001 |
| Inspiratory Resistance (mmHg/slm) | Aura Gain^TM^ | LT^®^evo | -0.03 (-0.34 to 0.45) | 1.0 |
| Inspiratory Resistance (mmHg/slm) | Aura Gain^TM^ | LTS-D | -0.07 (-0.37 to 0.24) | 0.893 |
| Inspiratory Resistance (mmHg/slm) | Aura Gain^TM^ | i-gel® Plus | -0.02 (-0.06 to 0.02) | 0.34 |
| Inspiratory Resistance (mmHg/slm) | LT^®^evo | i-gel® plus | -0.02 (-0.06 to 0.01) | 0.19 |
| Inspiratory Resistance (mmHg/slm) | LTS-D | i-gel® plus | -0.10 (-0.17 to -0.06) | <0.001 |

Abbreviation: slm = standard liters per minute; CI = confidence interval

# Supplement Table 2 – Sensitivity analysis for KS 2, 5, and 6 for inspiratory pressure, tidal volume, flow, and inspiratory resistance

| **Device** | **Tidal Volume [L]** | **Flow [slm]** | **Pressure [mmHg]** | **Resistance [mmHg/slm]** |
| --- | --- | --- | --- | --- |
| LT®evo | 0.37 (0.36–0.52) | 14.9 (14.2–16.6) | 11.6 (10.9–14.6) | 0.79 (0.77–0.85) |
| Aura Gain^TM^ | 0.37 (0.37–0.53) | 14.2 (13.2–17.0) | 11.2 (10.7–13.3) | 0.80 (0.77–0.84) |
| LTS-D | 0.38 (0.37–0.53) | 15.2 (14.5–16.9) | 10.7 (9.8–10.9) | 0.72 (0.58–0.75) |
| i-gel® Plus | 0.38 (0.37–0.52) | 14.6 (13.9–16.7) | 11.3 (10.9–15.4) | 0.82 (0.77–0.91) |

Values are presented in Median (Q1-Q3). Abbreviation: slm = standard liters per minute

# Supplement Table 3 – Sensitivity analysis for KS 2,5, and 6 Comparison of inspiratory pressure, tidal volume, and inspiratory resistance

| **Parameter** | **Device A** | **Device B** | **Median Difference (95% CI)** | **p-value** |
| --- | --- | --- | --- | --- |
| Tidal Volume (mL) | AuraGain™ | LT®evo | +0.007 (0.0002 to 0.012) | 0.040 |
| Tidal Volume (mL) | LT®evo | LTS-D | −0.009 (−.0.013 to −0.004) | 0.009 |
| Tidal Volume (mL) | AuraGain™ | LTS-D | −0.003 (−0.010 to 0.006) | 0.48 |
| Tidal Volume (mL) | i-gel® | LT®evo | +0.003 (−0.006 to 0.012) | 0.41 |
| Tidal Volume (mL) | i-gel® | AuraGain™ | + 0.001 (−0.008 to 0.010) | 0.81 |
| Tidal Volume (mL) | i-gel® | LTS-D | +0.0024 (−0.007 to 0.012) | 0.52 |
| Inspiratory Pressure (mmHg) | LT®evo | LTS-D | +0.96 (0.61 to 1.34) | <0.001 |
| Inspiratory Pressure (mmHg) | AuraGain™ | LTS-D | +0.58 (0.22 to 0.97) | 0.002 |
| Inspiratory Pressure (mmHg) | i-gel® | LTS-D | +0.63 (0.19 to 1.07) | 0.004 |
| Inspiratory Pressure (mmHg) | LT®evo | AuraGain™ | +0.38 (0.04 to 0.73) | 0.028 |
| Inspiratory Pressure (mmHg) | LT®evo | i-gel® | +0.34 (−0.02 to 0.71) | 0.071 |
| Inspiratory Pressure (mmHg) | i-gel® | AuraGain™ | +0.04 (−0.31 to 0.41) | 0.84 |
| Inspiratory Resistance (mmHg/slm) | LT®evo | LTS-D | +0.076 (0.056 to 0.105) | <0.001 |
| Inspiratory Resistance (mmHg/slm) | AuraGain™ | LTS-D | +0.083 (0.059 to 0.112) | <0.001 |
| Inspiratory Resistance (mmHg/slm) | i-gel® | LTS-D | +0.099 (0.061 to 0.169) | <0.001 |
| Inspiratory Resistance (mmHg/slm) | LT®evo | AuraGain™ | −0.007 (−0.031 to 0.018) | 0.61 |
| Inspiratory Resistance (mmHg/slm) | i-gel® | LT®evo | +0.023 (−0.012 to 0.061) | 0.19 |
| Inspiratory Resistance (mmHg/slm) | i-gel® | AuraGain™ | +0.016 (−0.021 to 0.057) | 0.34 |

Abbreviation: slm = standard liters per minute; CI = confidence interval
